# Supplementary material for: Resolving the full spectrum of human genome variation using Linked-Reads
Source: Genome Res. 2019 Apr;29(4):635–45. doi: 10.1101/gr.234443.118 (PMC6442396; doi:10.1101/gr.234443.118)
Supplement: Supplemental Material [file supp_gr.234443.118_Supplemental_longranger-2.2.zip › longranger-2.2.tar.bz2!/lr2.2-public/lib/stan/cmdstan/stan/doc/api/html/var_8hpp_source.html]

Stan: src/stan/agrad/rev/var.hpp Source File


|  |  |
| --- | --- |
|  | Stan  2.5.0  probability, sampling & optimization |


- Main Page
- Related Pages
- Namespaces
- Classes
- Files

- File List
- File Members

All Classes Namespaces Files Functions Variables Typedefs Enumerations Enumerator Friends Macros Pages

- src
- stan
- agrad
- rev

var.hpp

Go to the documentation of this file.

1 #ifndef STAN\_\_AGRAD\_\_REV\_\_VAR\_HPP

2 #define STAN\_\_AGRAD\_\_REV\_\_VAR\_HPP

3

4 #include <ostream>

5 #include <stan/math/matrix/Eigen.hpp>

6 #include <stan/agrad/rev/vari.hpp>

7

8 namespace stan {

9  namespace agrad {

10

11  // forward declare

12  static void grad(chainable\* vi);

13

27  class var {

28  public:

29

30  // FIXME: doc what this is for

31  typedef double Scalar;

32

40  vari \* vi\_;

41

51  bool is\_uninitialized() {

52  return (vi\_ == static\_cast<vari\*>(0U));

53  }

54

60  explicit var(vari\* vi)

61  : vi\_(vi)

62  { }

63

71  var()

72  : vi\_(static\_cast<vari\*>(0U))

73  { }

74

81  var(bool b) :

82  vi\_(new vari(static\_cast<double>(b))) {

83  }

84

91  var(char c) :

92  vi\_(new vari(static\_cast<double>(c))) {

93  }

94

101  var(short n) :

102  vi\_(new vari(static\_cast<double>(n))) {

103  }

104

111  var(unsigned short n) :

112  vi\_(new vari(static\_cast<double>(n))) {

113  }

114

121  var(int n) :

122  vi\_(new vari(static\_cast<double>(n))) {

123  }

124

131  var(unsigned int n) :

132  vi\_(new vari(static\_cast<double>(n))) {

133  }

134

141  var(long int n) :

142  vi\_(new vari(static\_cast<double>(n))) {

143  }

144

151  var(unsigned long int n) :

152  vi\_(new vari(static\_cast<double>(n))) {

153  }

154

161  var(unsigned long long n) :

162  vi\_(new vari(static\_cast<double>(n))) {

163  }

164

171  var(long long n) :

172  vi\_(new vari(static\_cast<double>(n))) {

173  }

174

181  var(float x) :

182  vi\_(new vari(static\_cast<double>(x))) {

183  }

184

190  var(double x) :

191  vi\_(new vari(x)) {

192  }

193

200  var(long double x) :

201  vi\_(new vari(static\_cast<double>(x))) {

202  }

203

209  inline double val() const {

210  return vi\_->val\_;

211  }

212

221  inline double adj() const {

222  return vi\_->adj\_;

223  }

224

237  void grad(std::vector<var>& x,

238  std::vector<double>& g) {

239  stan::agrad::grad(vi\_); // defined in chainable.hpp

240  g.resize(x.size());

241  for (size\_t i = 0; i < x.size(); ++i)

242  g[i] = x[i].vi\_->adj\_;

243  }

244

245  // POINTER OVERRIDES

246

259  inline vari& operator\*() {

260  return \*vi\_;

261  }

262

273  inline vari\* operator->() {

274  return vi\_;

275  }

276

277  // COMPOUND ASSIGNMENT OPERATORS

278

289  inline var& operator+=(const var& b);

290

301  inline var& operator+=(const double b);

302

314  inline var& operator-=(const var& b);

315

327  inline var& operator-=(const double b);

328

340  inline var& operator\*=(const var& b);

341

353  inline var& operator\*=(const double b);

354

365  inline var& operator/=(const var& b);

366

378  inline var& operator/=(const double b);

379

388  friend std::ostream& operator<<(std::ostream& os, const var& v) {

389  if (v.vi\_ == 0)

390  return os << "uninitialized";

391  return os << v.val() << ':' << v.adj();

392  }

393  };

394

395  }

396 }

397 #endif

stan::agrad::var::operator/=

var & operator/=(const var &b)

The compound divide/assignment operator for variables (C++).

**Definition:** operator\_divide\_equal.hpp:10

stan::agrad::var::operator-=

var & operator-=(const var &b)

The compound subtract/assignment operator for variables (C++).

**Definition:** operator\_minus\_equal.hpp:10

stan::agrad::var::adj

double adj() const

Return the derivative of the root expression with respect to this expression.

**Definition:** var.hpp:221

stan::agrad::var::operator->

vari \* operator->()

Return a pointer to the underlying implementation of this variable.

**Definition:** var.hpp:273

vari.hpp

stan::agrad::var::var

var(int n)

Construct a variable by static casting the specified value to double.

**Definition:** var.hpp:121

stan::agrad::var::val

double val() const

Return the value of this variable.

**Definition:** var.hpp:209

stan::agrad::vari::val\_

const double val\_

The value of this variable.

**Definition:** vari.hpp:37

stan::agrad::var::var

var(float x)

Construct a variable by static casting the specified value to double.

**Definition:** var.hpp:181

stan::agrad::var::var

var(long int n)

Construct a variable by static casting the specified value to double.

**Definition:** var.hpp:141

stan::agrad::grad

static void grad(chainable \*vi)

Compute the gradient for all variables starting from the specified root variable implementation.

**Definition:** chainable.hpp:110

stan::agrad::var::var

var(unsigned short n)

Construct a variable by static casting the specified value to double.

**Definition:** var.hpp:111

stan::agrad::var::operator\*

vari & operator\*()

Return a reference to underlying implementation of this variable.

**Definition:** var.hpp:259

stan::agrad::var::vi\_

vari \* vi\_

Pointer to the implementation of this variable.

**Definition:** var.hpp:40

stan::agrad::var::var

var(char c)

Construct a variable by static casting the specified value to double.

**Definition:** var.hpp:91

stan::agrad::var::operator\*=

var & operator\*=(const var &b)

The compound multiply/assignment operator for variables (C++).

**Definition:** operator\_multiply\_equal.hpp:10

stan::agrad::var::var

var(unsigned long int n)

Construct a variable by static casting the specified value to double.

**Definition:** var.hpp:151

stan::agrad::var::var

var(bool b)

Construct a variable by static casting the specified value to double.

**Definition:** var.hpp:81

stan::agrad::var::grad

void grad(std::vector< var > &x, std::vector< double > &g)

Compute the gradient of this (dependent) variable with respect to the specified vector of (independen...

**Definition:** var.hpp:237

stan::agrad::vari

The variable implementation base class.

**Definition:** vari.hpp:28

stan::agrad::var::var

var(short n)

Construct a variable by static casting the specified value to double.

**Definition:** var.hpp:101

stan::agrad::var::var

var(long long n)

Construct a variable by static casting the specified value to double.

**Definition:** var.hpp:171

stan::agrad::var::var

var(long double x)

Construct a variable by static casting the specified value to double.

**Definition:** var.hpp:200

stan::agrad::var::var

var(vari \*vi)

Construct a variable from a pointer to a variable implementation.

**Definition:** var.hpp:60

stan::agrad::var::var

var(unsigned int n)

Construct a variable by static casting the specified value to double.

**Definition:** var.hpp:131

Eigen.hpp

stan::agrad::var::is\_uninitialized

bool is\_uninitialized()

Return true if this variable has been declared, but not been defined.

**Definition:** var.hpp:51

stan::agrad::var::var

var(double x)

Construct a variable with the specified value.

**Definition:** var.hpp:190

stan::agrad::var

Independent (input) and dependent (output) variables for gradients.

**Definition:** var.hpp:27

stan::agrad::var::operator<<

friend std::ostream & operator<<(std::ostream &os, const var &v)

Write the value of this auto-dif variable and its adjoint to the specified output stream...

**Definition:** var.hpp:388

stan::agrad::var::var

var(unsigned long long n)

Construct a variable by static casting the specified value to double.

**Definition:** var.hpp:161

stan::agrad::var::Scalar

double Scalar

**Definition:** var.hpp:31

stan::agrad::var::operator+=

var & operator+=(const var &b)

The compound add/assignment operator for variables (C++).

**Definition:** operator\_plus\_equal.hpp:10

stan::agrad::vari::adj\_

double adj\_

The adjoint of this variable, which is the partial derivative of this variable with respect to the ro...

**Definition:** vari.hpp:43

stan::agrad::var::var

var()

Construct a variable for later assignment.

**Definition:** var.hpp:71


---

[ Stan Home Page ]

*© 2011–2014,
Stan Development Team.*
